# Die Auswirkungen von Primärdatenqualität und -interoperabilität auf Analysen von Real-World-Daten

**DOI:** 10.1007/s00103-023-03824-y

**Published:** 2024-01-04

**Authors:** Stefanie Weber

**Affiliations:** Abteilung „Kodiersysteme und Register“ (K), Bundesinstitut für Arzneimittel und Medizinprodukte, Kurt-Georg-Kiesinger-Allee 3, 53175 Bonn, Deutschland

**Keywords:** RWD, Semantische Standardisierung, Kodiersystem, Europäischer Gesundheitsdatenraum, EHDS, RWD, Semantic standardisation, Code system, European health data space, EHDS

## Abstract

Real-World-Daten rücken im Rahmen der Digitalisierung immer mehr in den Fokus der Versorgungsforschung. Die zeitnahe Verfügbarkeit von großen Datenmengen lässt hoffen, dass Forschungsfragen ohne zusätzliche Datenerhebung schnell beantwortet und ein direkter Nutzen für die Versorgung von Menschen erreicht werden kann. Gerade in akuten Versorgungslagen, wie Hitzewellen oder einer Pandemie, kann dies entscheidend sein. Doch hängen die Real-World-Daten ganz maßgeblich von der Qualität und Intention der Datenerhebung ab. Sie werden auch durch Festlegungen auf semantische und syntaktische Standards beeinflusst, die für Primärdaten getroffen werden – oft mit heterogenen Zielsetzungen. Im Rahmen der verschiedenen Initiativen auf nationaler wie auf internationaler Ebene sollten deshalb ein holistischer Blick auf Datenerhebung und Auswertung und ein regelhafter Rückkopplungsmechanismus zwischen Datenauswertung und Festlegungen für die Erhebung angestrebt werden. Durch eine Einbeziehung von Anforderungen an die sekundäre Datenauswertung in die Festlegungsprozesse für die Datenerhebung kann die Aussagekraft der Daten für die Forschung langfristig erhöht werden.

In diesem Diskussionsbeitrag werden zunächst die Aktivitäten zur standardisierten Datenerfassung im Rahmen der Digitalisierungsinitiativen und die entsprechenden europäischen Ansätze dargestellt. Anhand der Auswirkungen dieser Aktivitäten auf Möglichkeiten und Schwierigkeiten der Datenzusammenführung für Analysen von Real-World-Daten wird schließlich im Beitrag für einen anhaltenden Diskurs zwischen den verschiedenen Bereichen geworben.

## Abstract (en)

Real-world data is increasingly becoming the focus of healthcare research in the context of digitization. The timely availability of large amounts of data gives hope that research questions can be answered quickly without additional data collection and that a direct benefit for the care of people can be achieved. Especially in acute care situations, such as heat waves or a pandemic, this can be crucial. But real-world data depend quite significantly on the quality and intent of data collection. It is also influenced by determinations on semantic and syntactic standards that are made for primary data – often considering different use cases. In the context of different initiatives on national and international levels, a holistic view on data collection and evaluation and a regular feedback mechanism between data evaluation and specifications for the collection should be established. By including requirements for secondary data evaluation in the definition processes for data collection, the informative value of the data for research can be increased in the long term.

In this discussion paper, the activities for standardized data collection in the context of the digitization initiatives and the corresponding European approaches are first presented. After outlining the effects of these activities on the possibilities and difficulties of data consolidation for the analysis of real-world data, the article calls for an ongoing discourse between the different areas.

## Einleitung

Die Digitalisierung im Gesundheitswesen schreitet voran. Nicht nur in Deutschland wird mit großen Initiativen, wie z. B. dem Krankenhauszukunftsgesetz, in die Digitalisierung investiert. Zugleich wird große Hoffnung in die Forschung anhand von Real-World-Daten gesteckt. Sowohl die Digitalisierung als auch die Forschung sollen letztendlich der Bevölkerung zugutekommen und die Prävention, Diagnostik und Behandlung effizienter, sicherer und besser machen. Nicht immer wird dabei daran gedacht, dass die Digitalisierung der Versorgungsprozesse direkten Einfluss auf die Forschung anhand von Real-World-Daten haben kann. In diesem Diskussionsbeitrag sollen die im Rahmen der Digitalisierung erfolgende Standardisierung der Datenerhebung und Übermittlung beleuchtet, die Ansprüche an die Auswertungen von Real-World-Daten und das Wechselspiel der beiden Bereiche national als auch international betrachtet werden. Dafür werden die Begrifflichkeiten „Primärdaten“, „Sekundärdaten“ und „Real-World-Daten“ entsprechend der Tab. 1 definiert, da insbesondere der Begriff „Real-World-Daten“ in verschiedenen Zusammenhängen unterschiedlich benutzt wird [1, 2]. In den folgenden Abschnitten werden zuerst die standardisierte Datenerfassung und der entsprechende Blick auf die europäischen Ansätze dargestellt. Danach wird auf die Möglichkeiten und Schwierigkeiten der Datenzusammenführung geschaut und das Fazit als Diskussionsbeitrag zum anhaltenden Diskurs im Rahmen der Digitalisierungsinitiativen gezogen.

**Table 1:** 

Begriff	Definition im Kontext dieses Artikels
Primärdaten	Primärdaten sind Daten, die im Kontext der Gesundheitsversorgung für verschiedene Anwendungszwecke erhoben werden. Hierzu gehören unter anderem Abrechnungsdaten oder Verordnungsdaten
Sekundärdaten	Sekundärdaten sind Daten für eine Nutzung in einem anderen Kontext als dem, in dem sie ursprünglich erhoben wurden. Dies sind z. B. zusammengeführte Daten aus Abrechnung und Qualitätssicherung, die „sekundär“ z. B. für Forschungszwecke analysiert werden
Real-World-Daten	Real-World-Daten (RWD) sind Daten, die ein Kollektiv von Personen in realer Versorgungssituation darstellen sollen. Hierfür können verschiedene Primärdatenquellen verwendet und im Rahmen einer sekundären Datenanalyse zusammengeführt werden. Entsprechend der gängigsten Verwendung des Begriffes sollen auch in diesem Kontext nicht die randomisierten klinischen Studien (RCT) inkludiert sein, auch wenn auf diese grundsätzlich die Kernaussagen des Artikels zutreffen können

## Datenerfassung und -übermittlung im Kontext der Digitalisierung

Im Gesundheitswesen wird eine Vielzahl von Daten für die verschiedensten Zwecke erhoben. Neben der klinischen Dokumentation spielen rechtliche Aspekte, Qualitätssicherungs- und Abrechnungserfordernisse eine wichtige Rolle, um nur einige zu nennen. In der papierbasierten Dokumentation wurden viele Daten mehrfach erhoben oder mehrfach dokumentiert, was mit der zunehmenden Digitalisierung idealerweise verbessert wird. Aber Digitalisierung heißt nicht nur, die papiergebundenen Dokumentationen in ein digitales Format zu überführen. Vielmehr sollte Digitalisierung auch mit einem gründlichen Überdenken der zu erhebenden Inhalte, des Datenflusses und der Bearbeitungsschritte einhergehen. Wird beispielsweise die Kommunikation zwischen Labor und Station in einem Krankenhaus digitalisiert, so reicht es nicht, die bisherigen papiergebundenen Daten digital zu übermitteln. Vielmehr sollte gleich mit bedacht werden, welche dieser Daten in welche weiteren Systeme übernommen werden sollen, welche in die Kommunikation mit Dritten fließen und welche vielleicht auch in einem Verlauf über mehrere Jahre mit weiteren Labordaten aus anderen Quellen zusammengeführt werden könnten. Und an diesem Punkt kommt ein entscheidender Faktor ins Spiel: die Standardisierung der Daten. Neben den Schnittstellen zwischen Systemen müssen auch die Kommunikationsformate und Inhalte standardisiert werden. Die sogenannte syntaktische und semantische Standardisierung ist ein wesentlicher Faktor für eine Maschine-zu-Maschine-Kommunikation, die eine sinnvolle Weiterverwendung in dem empfangenden System und für weitere Anwendungszwecke erlaubt. Für das Beispiel der Labordaten gibt es eine Festlegung für die syntaktische und semantische Standardisierung: das Medizinische Informationsobjekt (MIO) „Laborbefund“ [3]. Idealerweise werden an allen Stellen, an denen Labordaten kommuniziert werden, in Zukunft die Festlegungen aus diesem MIO verwendet. Das bedeutet eine erhebliche Umstellung vieler Systeme, denn bisher wurden Labordaten in den verschiedenen Laboren und den nachgelagerten Systemen unterschiedlich abgespeichert. Viele Labore hatten eigene Wertelisten für die Ergebnisse, verwendeten unterschiedliche Einheiten und bezeichneten die Untersuchungen teilweise auch unterschiedlich. In der Entwicklung des MIO wurde nach intensiver fachlicher Diskussion eine Festlegung getroffen: Für die Kommunikation über Laborwerte werden vor allem die Kodiersysteme LOINC^1^, SNOMED CT^2^ und UCUM^3^ eine wichtige Rolle für die semantische Standardisierung spielen. Für die syntaktische Standardisierung ist die einheitliche Festlegung, den internationalen Standard HL7 FHIR^4^ zu verwenden [4], der vor Kurzem auch als zukünftiger Standard für den Datenaustausch in Europa im Rahmen der grenzübergreifenden Gesundheitsversorgung festgelegt wurde [5].

Außerdem spielt der Zweck der Datenerhebung eine entscheidende Rolle. Bereits mit der Verwendung eines einheitlichen Kodiersystems geht ein gewisses Risiko der Datenverzerrung einher, wenn es für unterschiedliche Zwecke verwendet wird. So ist bspw. die deutsche Modifikation der ICD-10, die ICD-10-GM (Internationale statistische Klassifikation der Krankheiten und verwandter Gesundheitsprobleme, 10. Revision, German Modification), in verschiedenen Anwendungen im Einsatz. Für die Kodierung im stationären Bereich spielt hier vor allem der Abrechnungszweck die entscheidende Rolle. Hierfür gibt es ein eigenes Regelwerk (Deutsche Kodierrichtlinien [6]), das zusätzlich zu den Kodierregeln der ICD-10-GM bestimmte Festlegungen trifft (z. B. die Haupt- und Nebendiagnosedefinition). Im ambulanten Sektor gibt es dieses Regelwerk nicht, weil hier die Grundlage für die Vergütung der einheitliche Bewertungsmaßstab für ärztliche Leistungen (EBM) ist, sodass es zu unterschiedlicher Kodierung kommen kann und die Qualität der kodierten Daten in Untersuchungen angezweifelt wird [7]. Das kann direkten Einfluss auf die wissenschaftliche Arbeit mit Sekundärdaten und deren Ergebnisse haben und muss bei den Auswertungen beachtet werden [8].

Auch sind in der Datenerhebung vielfach unstrukturierte Freitexte ein wesentlicher Teil, der sich für eine Standardisierung deutlich komplexer darstellt und bei der Angleichung verschiedener bisheriger Papierdokumentationen an ein digitales einheitliches Format zusätzliche Herausforderungen mit sich bringt [9].

Der Zweck der Datenerhebung ist auch bei Arzneimitteldaten ein wichtiger Faktor für die sekundäre Datenzusammenführung. Schaut man sich die Zulassungsprozesse für Arzneimittel an, so gibt es hierfür Festlegungen auf Kodiersysteme, im Prozess der Verordnung wiederum wurden andere Festlegungen getroffen. So ist in Deutschland die Pharmazentralnummer ein Beispiel eines Kodiersystems, das zwar sehr spezifische Informationen zu Warenzeichen, Wirkstoffstärke, Darreichungsform, Packungsgröße und pharmazeutischem Hersteller enthält, aber nur im ambulanten Sektor im Rahmen der Verordnung zur Anwendung kommt. Ein weiteres verwendetes Kodiersystem ist die weniger detailreiche, aber dafür strukturierte Anatomisch-Therapeutisch-Chemische (ATC) Klassifikation, die für die Sekundärdatenanalysen Auswertungen auf verschiedenen Hierarchieebenen ermöglicht. Und möchte man Nebenwirkungen eines Medikamentes dokumentieren, so erfolgt dies im Versorgungsbereich im Kontext der Diagnosedokumentation mithilfe des Kodiersystems ICD-10-GM, für den Bereich der Pharmakovigilanz wiederum mit dem Kodiersystem MedDRA^5^.

Schaut man sich das Beispiel von chirurgischen Eingriffen an, so werden diese in Deutschland vor allem mit dem Kodiersystem OPS^6^ verschlüsselt. Dieses Kodiersystem wird seit mehr als 20 Jahren in Deutschland verwendet und spielt vor allem für die Abrechnung von Leistungen eine entscheidende Rolle. OPS-Kodes sind z. B. sowohl im deutschen DRG^7^-System relevant als auch für das ambulante Operieren^8^. In jährlichen Zyklen wird das Kodiersystem weiterentwickelt und angepasst. Entsprechende Daten werden z. B. nach der Datenübermittlungsvereinbarung nach § 301 Abs. 3 SGB V zwischen Krankenhäusern und Krankenkassen kommuniziert. Schaut man nun aber in die MIO, so findet man dort zwar auch OPS-Kodes, es sind aber ebenfalls SNOMED-CT-Kodes für das Abbilden von Prozeduren hinterlegt, die in der Abrechnung bisher keine Rolle spielen. Doch warum wird in den MIO diese zusätzliche Kodiermöglichkeit vorgegeben?

## Der Blick nach Europa

**Figure 1:**
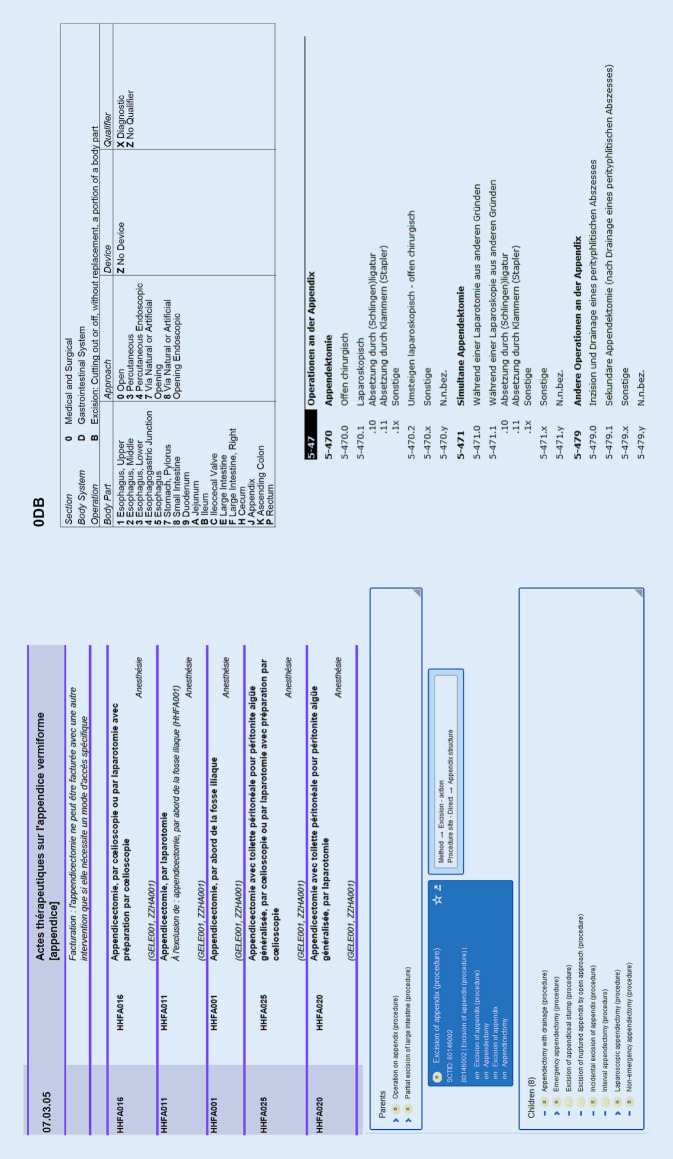

In Europa ist das Bild sehr heterogen, wenn man sich die Kodierung von chirurgischen Eingriffen ansieht. Viele Länder haben eigene Kodiersysteme entwickelt, einige setzen auch auf das US-amerikanische System ICD-10-PCS^9^ oder sogar noch auf dessen Vorgänger, die ICD-9-CM^10^ (Abb. 1). In den meisten Ländern steht wie auch in Deutschland der Anwendungszweck der Abrechnung im Vordergrund. Für statistische Auswertungen zu chirurgischen Eingriffen ist es schwer, die Daten aus den verschiedenen Ländern zu vergleichen, da die verschiedenen Kodiersysteme sehr unterschiedlich aufgebaut sind. Bereits das „Hospital Data Project“, das in den Jahren 2003 und 2008 statistische Vergleiche zwischen Krankenhausdaten aus Europa durchführte, hat für Operationen nur einen Vergleich auf sehr grober Basis durchführen können [10]. Damals verwendeten noch viele Länder die ICD-9-CM und noch heute stellt EUROSTAT die entsprechenden Statistiken^11^ gruppiert nach ICD-9-CM-Kodes auf sehr grober Basis bereit.

**Figure 2:**
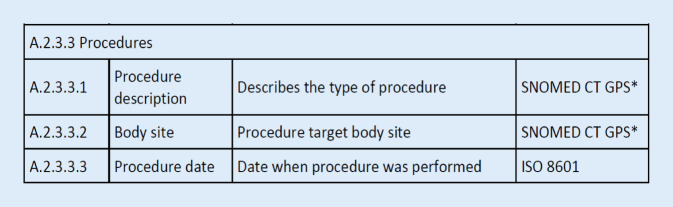

Was bedeutet das für den grenzübergreifenden Datenaustausch? Wenn Menschen reisen und an verschiedenen Orten eine medizinische Behandlung in Anspruch nehmen, so wäre ein Zugriff auf die versorgungsrelevanten Daten, wie z. B. Allergien, Implantate, aber auch vorangegangene Operationen oder dergleichen, hilfreich. Hierfür wurden entsprechende Strukturen auf europäischer Ebene geschaffen, die europäischen grenzübergreifenden Gesundheitsdienste MyHealth@EU^12^. Zur Nutzung dieser Strukturen ist auch eine Einigung auf europäische semantische und syntaktische Standardisierungen erforderlich. Für chirurgische Eingriffe hat man sich auf das Kodiersystem SNOMED CT geeinigt, allerdings bisher noch begrenzt auf die wichtigsten Kodes, die z. B. bei einer Notfallversorgung relevant sein könnten (Abb. 2).

Unter anderem deshalb findet sich im MIO „Patientenkurzakte“, also dem MIO, das die relevantesten Daten einer Person zusammenfasst, auch nicht nur das Kodiersystem OPS für Prozeduren, sondern auch das Kodiersystem SNOMED CT.

Mit der Planung des Europäischen Gesundheitsdatenraums (EHDS) werden bereits Vorbereitungen für die semantische und syntaktische Standardisierung getroffen. Bereits jetzt gibt es Guidelines, also Richtlinien, wie bestimmte Inhalte für den grenzübergreifenden Datenaustausch verwendet werden sollen. Für Europa sind dies die Guidelines des eHealth-Netzwerks [11]. Das eHealth-Netzwerk wurde basierend auf einem Beschluss des Europäischen Parlaments eingerichtet.^13^ In 6 Guidelines werden neben generellen Richtlinien die 5 Themenbereiche Patientenkurzakte, E‑Rezept, Labordaten, Bildgebung und Krankenhausentlassbrief adressiert. Aus den Ansätzen der Guideline Patientenkurzakte (Patient Summary) entstand ein internationaler Standard, der nun auch von ISO bereitgestellt und in Einklang mit den europäischen Überlegungen weiterentwickelt wird („International Patient Summary“ [IPS]^14^; Abb. 2).

Auch für die anfangs erwähnten Labordaten gibt es eine Festlegung. Diese Guideline wurde bei der Entwicklung des MIO „Laborbefund“ berücksichtigt und umgekehrt wurden auch die Überlegungen aus Deutschland in die europäische Richtlinie eingebracht, sodass eine Harmonisierung weitestgehend erfolgen konnte. Doch bis zu einer kompletten Harmonisierung der verwendeten Systeme in Europa braucht es Zeit, denn eine Umstellung ist nicht leicht, vor allem, wenn das jeweilige Kodiersystem bereits in vielen Anwendungen verwendet wird. So gibt es neben den Kodiersystemen für Labordaten, die in dem deutschen MIO festgelegt werden, ein weiteres Kodiersystem in Europa, das in mehreren Ländern genutzt wird: NPU^15^. In der Guideline zu Labordaten des eHealth-Netzwerks wird diese Thematik und auch die Möglichkeit eines sog. Mappings angesprochen.

Bei Arzneimitteln ist in Europa gerade eine Umstellung in Gange. Sowohl für die Zulassung als auch für die weitere Verwendung z. B. im Rahmen der ePrescription sollen in Zukunft Kodiersysteme entsprechend der Standardgruppe ISO IDMP^16^ verwendet werden. Die entsprechenden Entwicklungen laufen als großes europäisches Projekt [12], das die Zulassungsbehörden aller europäischen Länder beteiligt.

## Was bedeutet in diesem Zusammenhang Mapping?

Mapping kann automatisch, halbautomatisch und manuell erfolgen. Dabei werden die Inhalte des Kodes eines Kodiersystems auf einen entsprechenden Kode eines anderen Kodiersystems abgebildet, also quasi „übersetzt“. Wird also in Frankreich im Rahmen einer Behandlung mit der CCAM^17^, der französischen Prozedurenklassifikation, ein Kode für die Appendektomie angegeben, so kann dieser durch ein Mapping auf den entsprechenden Kode des OPS „übersetzt“ und die Information dann in den deutschen Systemen weiterverwendet werden, die Information zur vorangegangenen Behandlung steht den weiterbehandelnden Personen zur Verfügung. Beim automatischen Mapping ist es wichtig, dass jeweils nur eine „Übersetzungsmöglichkeit“ vorgegeben wird, also in beiden Kodiersystemen jeweils ein entsprechender Kode vorhanden ist. Ist dies nicht der Fall, so muss ggf. eine Vorentscheidung vom Erstellenden des Mappings getroffen werden. Oder man verwendet das Mapping halbautomatisch, bietet also bei der Umsetzung der Kodes bei einem entsprechenden Behandlungsfall dem Anwendenden die verschiedenen Auswahlmöglichkeiten an.

Schaut man sich das Beispiel der Appendektomie in Abb. 1 an, so sieht man in den verschiedenen Kodiersystemen, dass teilweise in den jeweiligen Kodes Inhalte angegeben werden, die in anderen Kodiersystemen nicht vorhanden sind, wie der Zugangsweg, die Frage, ob es sich um einen Notfalleingriff handelte, ob die Operation diagnostische Gründe hatte oder auch ob eine Peritonealspülung stattgefunden hat. Gibt das Mapping trotz nicht genau passender Inhalte für diese Fälle eine Festlegung vor, so kann es sein, dass Teile der Information verloren gehen, die bei der ursprünglichen Kodierung durch die behandelnde Person noch vorhanden waren.

Um diese Problematik zu umgehen, kann man die Datenerfassung durch ein möglichst detailliertes Kodiersystem vornehmen und dann in den jeweiligen Anwendungsszenarien auf das dafür erforderliche Kodiersystem „aggregieren“. Ein Beispiel für solch ein detailliertes Kodiersystem ist das Kodiersystem SNOMED CT. In Deutschland wurde dieses Kodiersystem in Hinblick auf die Initiativen zur Digitalisierung seit 2021 bereitgestellt. Die Übersetzung in die deutsche Sprache ist gestartet und die Implementierung in den Primärsystemen der Versorgung beginnt. Wird die Datenerfassung in einem gröberen System vorgenommen, so ist ein Mapping auf ein detaillierteres Kodiersystem zwingend mit Informationsverlust verbunden.

## Bedeutung für die Sekundärdatenanalyse

Die Analyse von Real-World-Daten muss alle diese Faktoren mit in Betracht ziehen. In den Auswertungen muss bedacht werden, welche Einflüsse auf die Datenerhebung eingewirkt haben. Ein Mapping, das im Bereich der Datenerhebung und -weitergabe zum Einsatz kommt, kann wesentlichen Einfluss auf die Sekundärdatenanalyse haben, insbesondere wenn Festlegungen im Mapping getroffen wurden, die eine systematische Verzerrung in den Daten bewirken können. Auch der Einfluss von zusätzlichen Regelwerken – wie z. B. den Deutschen Kodierrichtlinien – auf einen Teil der Versorgungsdaten muss berücksichtigt werden, auch in grenzübergreifenden Auswertungen.

**Figure 3:**
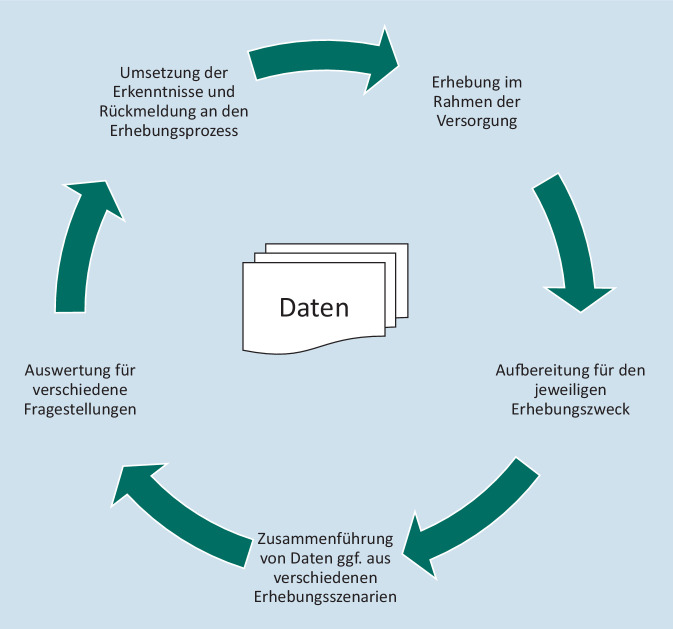

Die Auswertung von Daten, die im Rahmen der Versorgungsprozesse erhoben werden, können wichtige Erkenntnisse bringen, die wiederum verwendet werden können, um die Versorgungsprozesse zu verbessern (Abb. 3).

Für alle Versorgungsprozesse sollte man sich deshalb bei der Digitalisierung des Prozesses nicht nur die Fragen nach dem Datenfluss und der Datenstandardisierung für die Versorgung stellen. Vielmehr werden an diesem Punkt bereits entscheidende Weichen gestellt, die die Versorgungsforschung auf Real-World-Daten maßgeblich beeinflussen können. Im Hinblick auf die Entwicklung des Europäischen Gesundheitsdatenraums sollte bei den nationalen Überlegungen immer auch zusätzlich ein Abgleich mit europäischen Entwicklungen erfolgen. Schaut man sich das Beispiel der Labordaten an, so wird die Versorgungsforschung auf europäischer Ebene aktuell das Problem überwinden müssen, dass in Europa verschiedene Kodiersysteme verwendet werden. Möchte man Daten aus einem Land, das LOINC für die Kodierung verwendet, mit Daten aus einem anderen Land, das NPU für die Kodierung verwendet, zusammenführen, so muss ein Mapping erfolgen. Dies kann in großen Datenmodellen, wie z. B. OMOP^18^, hinterlegt werden. Geht aber mit dem Mapping ein Informationsverlust einher, so wird dieser die gesamte Datenauswertung betreffen. Für die Analyse wird das Kodiersystem mit der niedrigsten Detailtiefe als „kleinster gemeinsamer Nenner“ gewählt werden müssen. Möchte man Daten aus verschiedenen Ländern zusammenführen, die alle in wenig detaillierten Kodiersystemen erhoben wurden, so wird selbst ein Mapping der verschiedenen Kodiersysteme auf ein feiner granuläres Kodiersystem wie SNOMED CT keine detaillierten Analysen möglich machen. Durch den möglicherweise zusätzlichen und unterschiedlichen Informationsverlust des jeweiligen Mappings wird dieser im Zweifel sogar noch größer, als wenn ein direktes Mapping der beiden Kodiersysteme der Länder erfolgt. Möchte man aber in Europa Daten zusammenführen, die mit einer Vielzahl an unterschiedlichen Kodiersystemen erhoben wurden, wie aus dem Beispiel der Operationen in Abb. 1 ersichtlich, so kann das Mapping auf ein einheitliches Kodiersystem ggf. als einzige praktikable Lösung in Sekundärdatenauswertungen herangezogen werden.

Auch Large Language Models (LLMs) sind in diesem Kontext einsetzbar. Bei LLMs handelt es sich um Sprachmodelle, die mithilfe von künstlicher Intelligenz – trainiert auf einer sehr großen Datenmenge – helfen können Sprache zu verstehen oder zu übersetzen. Ihre primäre Stärke entfalten LLMs vor allem auf Rohdaten, also z. B. unstrukturiertem Text. Allerdings können sie auch, ggf. nach geeigneten Lerniterationen, die Erstellung oder den Einsatz eines Mappings unterstützen [13, 14]. Sind die Daten für den Versorgungsprozess bereits standardisiert worden, so werden auch durch den Einsatz der LLMs die unterschiedlichen Detailgrade der erhobenen Daten nicht überwunden werden können. Auch ist es für bestimmte Forschungsfragen essentiell, dass die verwendeten Mappings validiert und medizinisch korrekt sind. Insofern ist der alleinige Einsatz von LLMs zumindest kurz- und mittelfristig keine Option zur zufriedenstellenden Lösung des Problems der verschieden standardisierten Primärdaten, die in die Analyse der Sekundärdaten einfließen. Insofern stellt sich perspektivisch die Frage, ob die Stärke der LLMs nicht besser in der Datenerhebung genutzt werden könnte und die Datenerhebenden bei der Standardisierung der Daten unterstützt werden könnten. So können durch Einsatz von LLMs bei der Datenerhebung die Anwendenden bei der standardisierten Datenerhebung unterstützt und die Kodierung entsprechend den Gegebenheiten des jeweiligen Anwendungsfalls daraus abgeleitet werden. Der fertige standardisierte Datensatz muss dann durch den Anwendenden nur noch validiert werden.

Bei den Überlegungen zum Europäischen Gesundheitsdatenraum wird davon ausgegangen, dass viele Versorgungsdaten oder Registerdaten herangezogen werden können, um Real-World-Daten auszuwerten. In einem großen europäischen Projekt zur Vorbereitung des Europäischen Gesundheitsdatenraums, der Joint Action „Towards the European Health Data Space“ (TEHDAS), wurde die Interoperabilität von Daten untersucht [15]. Im Entwurf der Verordnung des europäischen Parlaments und des Rates über den europäischen Raum für Gesundheitsdaten^19^ findet sich das Kapitel IV zur Sekundärnutzung elektronischer Gesundheitsdaten. Hierin wird zwar auch auf die Notwendigkeit der Interoperabilität von Daten verwiesen, nicht jedoch in dem Umfang, in dem es in den vorherigen Abschnitten zur Primärdatenerhebung erfolgt. Nach der Verabschiedung wird in der Umsetzung der Verordnung weiter darauf hingewirkt werden müssen, dass der Ansatz des Datenzyklus auch im EHDS implementiert wird, und zwar insbesondere der Rückschluss von den Forschungsergebnissen auf Basis von Real-World-Daten zurück zu den Festlegungen für die Interoperabilität, die für die Primärdatenerhebung getroffen werden.

## Fazit

Die Erhebung von Daten für Versorgungsprozesse und die Auswertung von Daten für die Forschung sollten Hand in Hand gehen. Sowohl im nationalen als auch im internationalen Kontext kann ein gemeinsamer Ansatz für Datenerhebung und -auswertung die Aussagekraft deutlich erhöhen und somit ein größerer Nutzen für die Gesundheitsversorgung erreicht werden. Auch wenn die Umstellung der Datenerhebung zur Erfüllung der Anforderungen der Digitalisierung bereits alle Beteiligten vor große Herausforderungen stellt, darf in diesem Kontext die Anforderung der Sekundärdatennutzung nicht in den Hintergrund treten, wenn man die Aussagekraft der Ergebnisse von Sekundärdatenforschung verbessern möchte. Konsequenterweise muss immer wieder der Rückschluss zwischen den einzelnen Stationen der Daten hergestellt werden. Lässt man bei der Festlegung der Standards für die Datenerhebung und -weitergabe die Ergebnisse der Analyse der Real-World-Daten und insbesondere der dabei auftretenden Schwierigkeiten und Limitationen der Daten aus den Augen, so werden langfristig Chancen für einen kontinuierlichen Verbesserungsprozess der Datenqualität verpasst. Auch wenn zum jetzigen Zeitpunkt noch die Überwindung der heterogenen Standardisierungslandschaft in Deutschland und auch in Europa die größte Hürde für eine detaillierte Datenanalyse darstellt, so besteht perspektivisch durch die Initiativen im Bereich der Standardisierung der Datenerhebung deutliches Potenzial. Durch eine gemeinsame Betrachtung der Anforderungen an die Standardisierung von Versorgungsprozessen und der Forschungsfragen, die man durch die Analyse von Real-World-Daten lösen möchte, kann schon frühzeitig ein möglichst hoher Nutzen der Digitalisierung für beide Bereiche initialisiert werden. Selbst wenn dieser Prozess zum jetzigen Zeitpunkt zusätzlichen Abstimmungsaufwand mit sich bringt, können mittel- und langfristig dadurch auch eine Reduktion von nötigen Ressourcen für Forschungsprojekte erzielt und schnellere und validere Ergebnisse erreicht werden.

## Footnotes

1 Logical Observation Identifiers Names and Codes; https://loinc.org/; (Zugriff am 28.10.2023).

2 Systematized Nomenclature of Medicine, Clinical Terms; https://www.snomed.org/; (Zugriff am 28.10.2023).

3 Unified Code for Units of Measure; https://ucum.org/; (Zugriff am 28.10.2023).

4 Fast Healthcare Interoperability Resources; https://www.hl7.org/fhir/; (Zugriff am 28.10.2023).

5 Medical Dictionary for Regulatory Activities; https://www.meddra.org/; (Zugriff am 28.10.2023).

6 Operationen- und Prozedurenschlüssel; https://www.bfarm.de/DE/Kodiersysteme/Klassifikationen/OPS-ICHI/OPS/_node.html; (Zugriff am 28.10.2023).

7 Diagnosis Related Groups; https://www.g-drg.de/; (Zugriff am 28.10.2023).

8 Anhang 2 des EBM zum ambulanten Operieren; https://www.kbv.de/html/anhang_2.php; (Zugriff am 28.10.2023).

9 International Classification of Diseases, Tenth Revision, Procedure Coding System; https://www.cms.gov/medicare/icd-10/2023-icd-10-pcs; (Zugriff am 28.10.2023).

10 International Classification of Diseases, Ninth Revision, Clinical Modification (ICD-9-CM); https://www.cdc.gov/nchs/icd/icd9cm.htm; (Zugriff am 28.10.2023).

11 Surgical Operations and Procedures Statistics; EUROSTAT; https://ec.europa.eu/eurostat/statistics-explained/index.php?title=Surgical_operations_and_procedures_statistics#Number_of_surgical_operations_and_procedures; (Zugriff am 28.10.2023).

12 https://health.ec.europa.eu/ehealth-digital-health-and-care/electronic-cross-border-health-services_de ; (Zugriff am 28.10.2023).

13 DIRECTIVE 2011/24/EU OF THE EUROPEAN PARLIAMENT AND OF THE COUNCIL of 9 March 2011 on the application of patients’ rights in cross-border healthcare; https://eur-lex.europa.eu/legal-content/EN/TXT/?uri=celex:32011L0024.

14 ISO 27269: Health informatics – International patient summary; https://www.iso.org/standard/79491.html; (Zugriff am 28.10.2023).

15 Nomenclature for Properties and Units; https://www.npu-terminology.org/; (Zugriff am 28.10.2023).

16 Ausführung der European Medicine Agency zur Verwendung von ISO IDMP (International Organization for Standardization, Identification of medicinal products); https://www.ema.europa.eu/en/human-regulatory/overview/data-medicines-iso-idmp-standards-overview; (Zugriff am 28.10.2023).

17 Classification commune des actes médicaux; https://www.atih.sante.fr/les-versions-de-la-ccam; (Zugriff am 28.10.2023).

18 Observational Medical Outcomes Partnership (OMOP) Common Data Model; https://ohdsi.github.io/CommonDataModel/; (Zugriff am 28.10.2023).

19 https://eur-lex.europa.eu/legal-content/DE/TXT/HTML/?uri=CELEX:52022PC0197; (Zugriff am 23.08.2023).
